# La lutte contre la COVID-19 au Cameroun nécessite un second souffle

**DOI:** 10.11604/pamj.supp.2020.37.1.23535

**Published:** 2020-09-18

**Authors:** Jean Valentin Fokouo Fogha, Jean Jacques Noubiap

**Affiliations:** 1 Service d´Orl et Chirurgie Cervico-Faciale, Hôpital Régional de Bertoua, Bertoua, Cameroun,; 2 Centre de Prise en Charge de la COVID-19, Hôpital Régional de Bertoua, Bertoua, Cameroun,; 3 Médecins du Cameroun (MedCamer), Yaoundé, Cameroun,; 4 Centre for Heart Rhythm Disorders, University of Adelaide and Royal Adelaide Hospital, Adelaide, Australia

**Keywords:** Coronavirus, COVID-19, Cameroun, Coronavirus, COVID-19, Cameroon

## Abstract

La pandémie à coronavirus SARS-CoV-2 a été déclarée par l´Organisation Mondiale de la Santé le 11 janvier 2020. Actuellement, début mai 2020, elle a officiellement atteint plus de 3 millions de personnes à travers le monde, tuant environ 230 000 d´entre elles. Le Cameroun s´est progressivement organisé pour une riposte centrée sur des centres de prise en charge isolés des hôpitaux avec des fortunes diverses. Alors que la bataille contre ce virus fait rage, on peut percevoir quelques signes d´essoufflement, pourtant la pandémie risque de durer de nombreux mois encore. Cet article fait une analyse de situation et propose quelques solutions pour contribuer à endiguer l´actuelle pandémie et améliorer le système de gestion des urgences de santé publique au Cameroun.

## Abstract (en)

The coronavirus 2019 (COVID-19) pandemic was declared by the World Health Organization on January 11, 2020. As of early May 2020, the disease has affected more than 3 million people worldwide, killing around 230.000 of them. In Cameroon, the response to the COVID-19 pandemic has been gradually organized, with a strategic emphasis on dedicated treatment centers in the referral hospitals of the main cities. As the fight against the pandemic rages on, signs of running out of steam are becoming manifest, yet the pandemic is likely to last for many more months. This article analyzes the situation and suggests some solutions to help stem the current health crisis and improve the public health emergency management system in Cameroon.

## Perspectives

Le premier cas d´infection au SARS-CoV-2 (COVID-19) a été enregistré au Cameroun le 6 mars 2020. Au 2 mai 2020, on dénombre officiellement 2069 cas et 61 décès. Cependant, selon de nombreuses sources, les chiffres seraient largement sous-estimés [1,2]. Au début concentrée à Yaoundé et Douala, la pandémie touche désormais la totalité des dix régions du pays. La riposte s´est organisée en quatre axes: la recherche active et précoce des cas par un dépistage généralisé, la prise en charge des cas avec extension des capacités, la régulation sociale pour éviter la propagation, et la gouvernance et la redevabilité [3]. Sur le terrain, le caractère nouveau du COVID-19 et la reconfiguration des équipes opérationnelles de lutte contre les épidémies entraînent des lenteurs dues au temps d´adaptation et de formation des nouvelles équipes. Les difficultés logistiques et le manque d´équipements de protection viennent grever ce tableau peu reluisant. Certes, des efforts louables ont été faits: création des centres de prise en charge, décentralisation du test biologique de détection du SARS-CoV-2, dotation des régions en médicaments dont l´hydroxychloroquine et l´azithromycine, mise en place des Systèmes Régionaux de Gestion des Incidents (SGI), sensibilisation des populations et différents acteurs de la riposte, implémentation du lavage des mains et du port du masque dans l´espace public. Cependant, beaucoup reste à faire, d´autant plus que cette pandémie COVID-19 pourrait, selon plusieurs experts, durer plus d´une année avant d´être endiguée. Ainsi, de nouvelles perspectives devraient s´ouvrir dans cette lutte au Cameroun, et similairement dans plusieurs pays Africains.

**Prise en charge du personnel soignant:** dans un premier temps, il faudrait créer un cinquième axe de riposte, la prise en charge du personnel. Cet axe comprendrait la prise en charge psychologique, médicale et financière des personnes directement impliquées dans la lutte contre la COVID-19: médecins, pharmaciens, infirmiers, aides-soignants, hygiénistes, brancardiers, techniciens en maintenance biomédicale, chauffeurs, cuisiniers, secrétaires, informaticiens, etc. En effet, les personnels directement en contact avec les cas suspects et les patients COVID-19 sont soumis à un stress intense lié au risque de contamination qu´ils encourent tous les jours. Un barème uniforme sur toute l´étendue du pays doit être établi pour une prime de risque et une motivation financière de ces personnes. Le quatrième axe, gouvernance et redevabilité, signifie que les gestionnaires des affaires publiques doivent le faire dans l´éthique et l´équité, au profit des gouvernés et leur en rendre compte. La transparence et la justice sont donc inhérentes à ces deux concepts. En effet, la prise en charge multiforme des acteurs de première ligne de cette lutte contre la COVID-19 varierait significativement d´un centre à un autre, et d´une région à une autre. La disparité actuelle entretient un climat de suspicion et un sentiment d´injustice susceptibles de démotiver les acteurs sur le terrain. Dans ce contexte, des mouvements d´humeur sont à craindre dans l´avenir.

**Allocation et utilisation rationnelle des ressources disponibles:** ensuite, il faudrait utiliser rationnellement les ressources disponibles, notamment les tests de dépistage et les médicaments. Par exemple, le 21 avril dernier, le Ministre de la santé publique, à travers la note de service D31-170/NS/MINSANTE/SG/DPM, mettait à la disposition une première dotation de 429 à 1886 boîtes de 14 comprimés d´hydroxycloroquine 100 mg, 100 à 470 boîtes de 12 comprimés d´azithromycine 250 mg par région, comme ressource en médicaments pour une durée indéterminée. Si on s´en réfère au protocole national, ceci représente le traitement de 143 à 628 personnes pour la chloroquine et 200 à 940 personnes pour l´azithromycine, pour chacune des dix régions du pays. Dans la même période, la décentralisation des tests devenait effective, avec une dotation d´environ 1000 tests par région en dehors des capitales. Le constat pour l´instant est que la psychose créée par la pandémie au sein des populations augmente la demande de tests dont beaucoup ne sont pas justifiables. Le screening des personnes asymptomatiques, surtout dans un contexte de pénurie de tests n´est pas raisonnable. Si l´épidémie empire, on pourrait en manquer pour les cas symptomatiques, voire sévères. On pourra tester massivement lorsqu´il y aura plus de tests disponibles; mais pour le moment, cet examen devrait (re)devenir un acte subordonné à une prescription médicale du Centre de Prise en Charge ou des Equipes d´Intervention et d´Investigation Rapide (EIIR). A cet effet, un algorithme national doit être établi pour faire loi. Pour pallier aux ruptures multiples de médicaments et autres intrants, l´on devrait consentir des investissements financiers importants pour la riposte à la COVID-19. En effet, les équipements et consommables d´investigation, de tests, de soins et de réanimation manquent cruellement sur le terrain. Il faudrait mettre à contribution l´industrie locale pour ce qui peut être fait sur place. Ceci pour réduire le coût et limiter la fuite des devises qui sont déjà rares d´une part, et d´autre part, soutenir les Petites et Moyennes Industries/Entreprises (PMI/PME) qui suffoquent depuis le début de la crise sanitaire du fait de nombreuses restrictions.

**Protection du personnel soignant:** par ailleurs, le personnel soignant doit être protégé. De nombreuses contaminations ont déjà été enregistrées chez les soignants, dont certaines fatales. Il n´y a pas de statistiques officielles sur le nombre de soignants infectés. Mais selon le journal Le Messager, le 28 avril 2020 on dénombrait déjà 25 médecins contaminés dont 3 décès [4]. Au demeurant, le nombre d´infirmiers contaminés serait plus élevé que celui des médecins, car ils sont les plus en contact avec les malades. Les soldats de la santé ne sauraient remporter la bataille contre COVID-19 sans des armes adéquates. Sur le terrain, les efforts faits dans ce sens sont encore trop parcellaires, trop lents. Les besoins exprimés tardent à être satisfaits. Et pourtant, sans attendre, ces hommes et femmes doivent administrer des soins divers à ceux qui souffrent ou pourraient souffrir de la COVID-19. Il est donc impérieux d´apporter une dotation suffisante de ces équipements, non seulement aux centres dédiés de prise en charge, mais aussi à toutes les autres formations sanitaires du pays. Ces dernières doivent au minimum disposer d´une salle de tri où tous les patients sont reçus par un personnel disposant d´un équipement adéquat (sur blouse, visière, masque chirurgical, gants, solution hydroalcoolique, thermomètre laser), sommairement examinés à la recherche d´une possible infection au coronavirus, avant d´être orientés vers la consultation ordinaire ou vers un référent COVID-19. Les centres dédiés à la prise en charge des patients COVID-19, outre les traitements, doivent disposer de suffisamment d´équipements de protection individuelle (EPI). Actuellement, sur le terrain, il manque de tout, notamment de masques FFP2/FFP3/N95, obligeant les soignants à s´en procurer à leurs frais, à utiliser des moyens de bord ou à se lancer dans de dangereuses pratiques de recyclage non encadrées et qui ne garantissent pas leur sécurité. Il serait souhaitable que les personnels des centres de prise en charge soient testés périodiquement en vue de détecter précocement une infection et éviter la contamination de leurs familles et proches, ainsi que de leurs collègues et des malades.

**Sensibilisation communautaire:** il est également important de renforcer la sensibilisation communautaire sur le respect des mesures barrières. L´assouplissement des mesures visant à encourager la distanciation et un confinement volontaire (port obligatoire du masque, interdiction des rassemblements de plus de 50 personnes, fermeture des restaurants, boutiques et bars à 18 heures, etc.) prescrites par le gouvernement Camerounais a été annoncé le 30 avril dernier, à un moment où la pandémie se répand encore sur le territoire national et où des experts estiment que le pic de l´épidémie n´a pas encore été atteint. Cela a provoqué au sein des corps soignants de l´incompréhension, de la colère et pas loin du découragement. Ces mesures ont été prises pour des motifs économiques en raison de la grande prédominance du secteur informel animé par des personnes qui gagnent leur vie au jour le jour d´une part, et de la grande souffrance des PME/PMI du fait de la crise d´autre part. Pour un confinement efficace, les ménages et les entreprises auraient dû être soutenus financièrement. Devant cette situation, si d´autres mesures urgentes et fortes pour renforcer le système de santé et la riposte contre le Covid-19 ne sont pas prises, une catastrophe sanitaire se profile à l´horizon. Il faudrait organiser une communication de masse pour expliquer aux populations la nécessité de continuer à respecter les mesures barrières. Sur le terrain, en effet, les citoyens assimilent cet assouplissement à une fin de l´épidémie. Les bars et discothèques ont été envahis, et les masques et mesures barrières abandonnés par la plupart. Dans cette communication, un accent devrait être mis sur la stigmatisation que subissent injustement les patients COVID-19 et leurs soignants. Cette dernière empêche les patients de recourir à l´aide de l´hôpital. De leur cachette dans la communauté, ils disséminent ainsi la maladie. Un déficit ou une incohérence dans la communication en ce moment aura des conséquences désastreuses qui pourraient requérir un (re)confinement à la hâte.

**Décentralisation de la gestion de la crise:** il est impératif d´augmenter l´implication des collectivités territoriales décentralisées dans le système sanitaire. La COVID-19 pourrait être une opportunité d´accélérer la décentralisation comme le veulent les pouvoirs publics et le réclament les populations depuis de nombreuses années. Voulue par la Constitution de 1996, cette décentralisation tarde à être implémentée. C´est en décembre 2019 que la loi y relative a été votée [5]. Le transfert de pouvoir vers les mairies est lent et n´est pas suivi du transfert des ressources conséquentes. Pourtant, il leur revient désormais de recruter les personnels, les payer, encadrer les formations sanitaires de leur ressort territorial, inhumer les morts pendant les épidémies. Permettre aux collectivités territoriales décentralisées de jouer leur rôle en ce moment rendrait plus efficace et rapide la réponse à l´épidémie. Il faudrait rendre disponibles dans les centres de traitement tous les médicaments et dispositifs médicaux nécessaires au diagnostic et au traitement car la prise en charge des patients se veut gratuite. A cet effet, il serait souhaitable d´autonomiser financièrement les centres de prise en charge ou les hôpitaux de tutelle de ces centres.

**Regard vers l´avenir (renforcement du système de santé):** enfin, cette crise sanitaire devrait être une opportunité pour poser les jalons d´un système de santé plus performant. En effet, le modèle de la santé hospitalo-centré et hypercentralisé montre actuellement ses limites. Tout doit être repensé. De la formation initiale des professionnels de santé, à celle des gestionnaires des communes aux questions de santé, en passant par les transports, l´alimentation, les finances, les adductions d´eau et d´électricité entre autres, qui sont autant de secteurs imbriqués à celui de la santé. Les secteurs industriel et pharmaceutique doivent être redynamisés en vue de réduire notre dépendance vis-à-vis de l´extérieur. Il faudrait pouvoir produire localement au moins les antibiotiques les plus essentiels, les médicaments contre paludisme, la tuberculose et le VIH. La plupart de ces médicaments sont déjà hors brevet. Or ces pathologies sont celles qui sévissent le plus dans le pays et grèvent le budget de la santé par la commande des moyens de lutte sur le marché international. Même l´architecture des hôpitaux doit être revue et harmonisée pour faciliter la circulation des patients et des soignants en temps ordinaire ou d´épidémie. Cette crise sanitaire est l´occasion de penser à reset, une chance de faire mieux à l´avenir. Nous devons la saisir.

**Valorisation de la pharmacopée traditionnelle:** en ce début de mois de Mai 2020, il n´existe toujours pas de traitement curatif établi contre la COVID-19. L´antirétroviral lopinavir-ritonavir inclus dans certains protocoles de traitement au début de la pandémie n´a finalement pas montré d´efficacité dans les formes sévères de COVID-19 dans une étude randomisée contrôlée [6]. L´association hydroxychlorquine-azithromycine utilisée dans de nombreux pays a été recommandée notamment sur la base d´une étude clinique très contestée, du fait de ses limites méthodologiques majeures [7]. Depuis lors, plusieurs études ont remis en cause l´efficacité de l´hydroxychloroquine dans le traitement de la COVID-19 [8]. Le bénéfice potentiel de l´antiviral remdesivir suggéré dans une étude multinationale de faible échantillon [9] n´a pas été retrouvé dans une étude clinique randomisée contrôlée en Chine [10].

Face à cette absence de thérapie suffisamment efficace, plusieurs pays Africains ont choisi de miser sur des solutions thérapeutiques puisées dans la pharmacopée traditionnelle. L´exemple le plus populaire en date vient de Madagascar, où le Président de la République a présenté un produit à base de plantes dont l´Artemisia, et clamé son efficacité [11]. Ce produit, la COVID-Organics, a été massivement distribué aux populations malgaches, y compris aux enfants, essentiellement à visée prophylactique. Plusieurs pays Africains ont marqué leur adhésion à ce potentiel médicament, malgré la désapprobation de plusieurs organisations scientifiques et sanitaires dont l´Organisation Mondiale de la Santé [12,13]. En effet, le développement du Covid-Organics n´aurait pas été subordonné aux exigences scientifiques en la matière. Au Cameroun, un prélat de l´Eglise Catholique ayant plusieurs décennies d´expérience dans l´utilisation de plantes médicinales pour le traitement de diverses maladies, a annoncé la mise sur pied d´un produit phytothérapeutique contre la COVID-19 et débuté sa distribution [14]. Si ces démarches en marge des standards de recherche scientifique sont critiquables, notamment du fait des risques potentiels de ces produits qui n´ont pas été exclus par une recherche appropriée, les initiatives en elles-mêmes sont à saluer. En ce sens, il est important pour les Africains de rechercher des solutions locales à leurs problèmes de santé dont l´actuelle pandémie. Pour se faire, il est crucial que les tradipraticiens et les chercheurs universitaires travaillent ensemble pour apporter des médicaments de pharmacopée traditionnelle contre la COVID-19, après avoir prouvé, par des études cliniques bien menées, leur efficacité et leur innocuité.

## Conclusion

La pandémie au la COVID-19 pourrait encore durer de nombreux mois. La stratégie de lutte actuelle au Cameroun doit être réajustée. Ceci devrait inclure l´amélioration de la prise en charge du personnel, l´équipement adéquat des centres de traitement et l´allocation de plus grandes ressources, la décentralisation effective de la gestion de la riposte, et une sensibilisation accrue des populations. Aussi, cette crise sanitaire majeure devrait poser les jalons d´une restructuration profonde du système de santé et d´une amélioration à long terme de la gestion des urgences de santé publique au Cameroun. Enfin, la valorisation de la pharmacopée traditionnelle locale devrait constituer un axe de lutte important.
